# Targeting transcription factor TCF4 by γ-Mangostin, a natural xanthone

**DOI:** 10.18632/oncotarget.27159

**Published:** 2019-09-24

**Authors:** Balaji Krishnamachary, Dharmalingam Subramaniam, Prasad Dandawate, Sivapriya Ponnurangam, Pugazhendhi Srinivasan, Prabhu Ramamoorthy, Shahid Umar, Sufi Mary Thomas, Animesh Dhar, Seth Septer, Scott J. Weir, Thomas Attard, Shrikant Anant

**Affiliations:** ^1^Department of Cancer Biology, University of Kansas Medical Center, Kansas City, KS, USA; ^2^Department of General Surgery, University of Kansas Medical Center, Kansas City, KS, USA; ^3^Department of Otolaryngology, University of Kansas Medical Center, Kansas City, KS, USA; ^4^Department of Pediatrics, Division of Gastroenterology, Hepatology and Nutrition, University of Colorado, Aurora, CO, USA; ^5^Department of Pediatrics, Division of Gastroenterology, Children’s Mercy Hospital, Kansas City, KS, USA

**Keywords:** Wnt signaling, beta-catenin, natural product, tumor xenograft, preventive agent

## Abstract

Given that colon cancer is the third most common cancer in incidence and cause of death in the United States, and current treatment modalities are insufficient, there is a need to develop novel agents. Towards this, here we focus on γ-Mangostin, a bioactive compound present in the Mangosteen (*Garcinia mangostana*) fruit. γ-Mangostin suppressed proliferation and colony formation, and induced cell cycle arrest and apoptosis of colon cancer cell lines. Further, γ-Mangostin inhibited colonosphere formation. Molecular docking and CETSA (Cellular thermal shift assay) binding assays demonstrated that γ-Mangostin interacts with transcription factor TCF4 (T-Cell Factor 4) at the β-catenin binding domain with the binding energy of -5.5 Kcal/mol. Moreover, γ-Mangostin treatment decreased TCF4 expression and reduced TCF reporter activity. The compound also suppressed the expression of Wnt signaling target proteins cyclin D1 and c-Myc, and stem cell markers such as LGR5, DCLK1 and CD44. To determine the effect of γ-Mangostin on tumor growth *in vivo*, we administered nude mice harboring HCT116 tumor xenografts with 5 mg/Kg of γ-Mangostin intraperitoneally for 21 days. γ-Mangostin treatment significantly suppressed tumor growth, with notably lowered tumor volume and weight. In addition, western blot analysis revealed a significant decrease in the expression of TCF4 and its downstream targets such as cyclin D1 and c-Myc. Together, these data suggest that γ-Mangostin inhibits colon cancer growth through targeting TCF4. γ-Mangostin may be a potential therapeutic agent for colon cancer.

## INTRODUCTION

Current treatment modalities for colon cancer include surgery, radiation and chemotherapy; however, limitations include severe side effects and emergence of resistance. Therefore, there is a need of a novel therapeutic agent that targets in colon cancer. Wnt/β-catenin signaling is important in embryonic development and tissue homeostasis [1, 2]. In addition, the pathway is frequently dysregulated in cancers leading to high levels of expression of target genes. β-catenin, the key factor in the pathway is regulated by the multiprotein destruction complex consisting of multiple proteins including adenomatous polyposis coli (APC), axin, glycogen synthase kinase 3β (GSK3β), casein kinase 1α (CSK1α) and β-transducin repeats containing protein (β-TrCP). In the absence of a Wnt signal, β-catenin is phosphorylated by casein kinase 1α (CSK1α) and APC/Axin/GSK3-β complex and subsequently undergoes ubiquitination and proteasomal degradation. However, in the presence of Wnt ligand, receptor engagement relocalizes the destruction complex to the cell membrane wherein, the ubiquitination of phosphorylated β-catenin is blocked within the intact complex. Consequently, the complex becomes saturated by the phosphorylated form of β-catenin, leading to accumulation of newly synthesized β-catenin, free to translocate to the nucleus and to activate target genes [3, 4].

In colon cancer, the APC gene is frequently mutated generating in a truncated APC product that results in constitutive β-catenin activation. In addition, β-catenin mutations have been identified that result in its hyperactivation. This ultimately results in aberrant accumulation of stabilized β-catenin, followed by nuclear translocation and subsequent interaction with TCF4 (T-cell factor 4). The β-catenin:TCF4 complex plays a vital role in the canonical Wnt signaling pathway and thereby results in the activation of downstream targets such as cyclin D1 and c-myc. These genes play a significant role in cell proliferation, migration and survival. The inhibitors which target the upstream targets of the Wnt/β-catenin pathway fail to inhibit downstream APC and β-catenin mutation-driven gene transcriptions [5–8]. Inhibitors targeting the interaction between β-catenin, APC or E- cadherin may result in aberrant Wnt/β-catenin signaling that results in the formation of new cancer [9]. Selectively targeting the β-catenin-TCF4 interaction will not interfere with β-catenin/E-cadherin and β-catenin/APC interaction. Thus, targeting β-catenin-TCF4 interaction will have a significant impact on tumor cytotoxicity while remaining non-toxic to normal cells.

**Figure 1:**
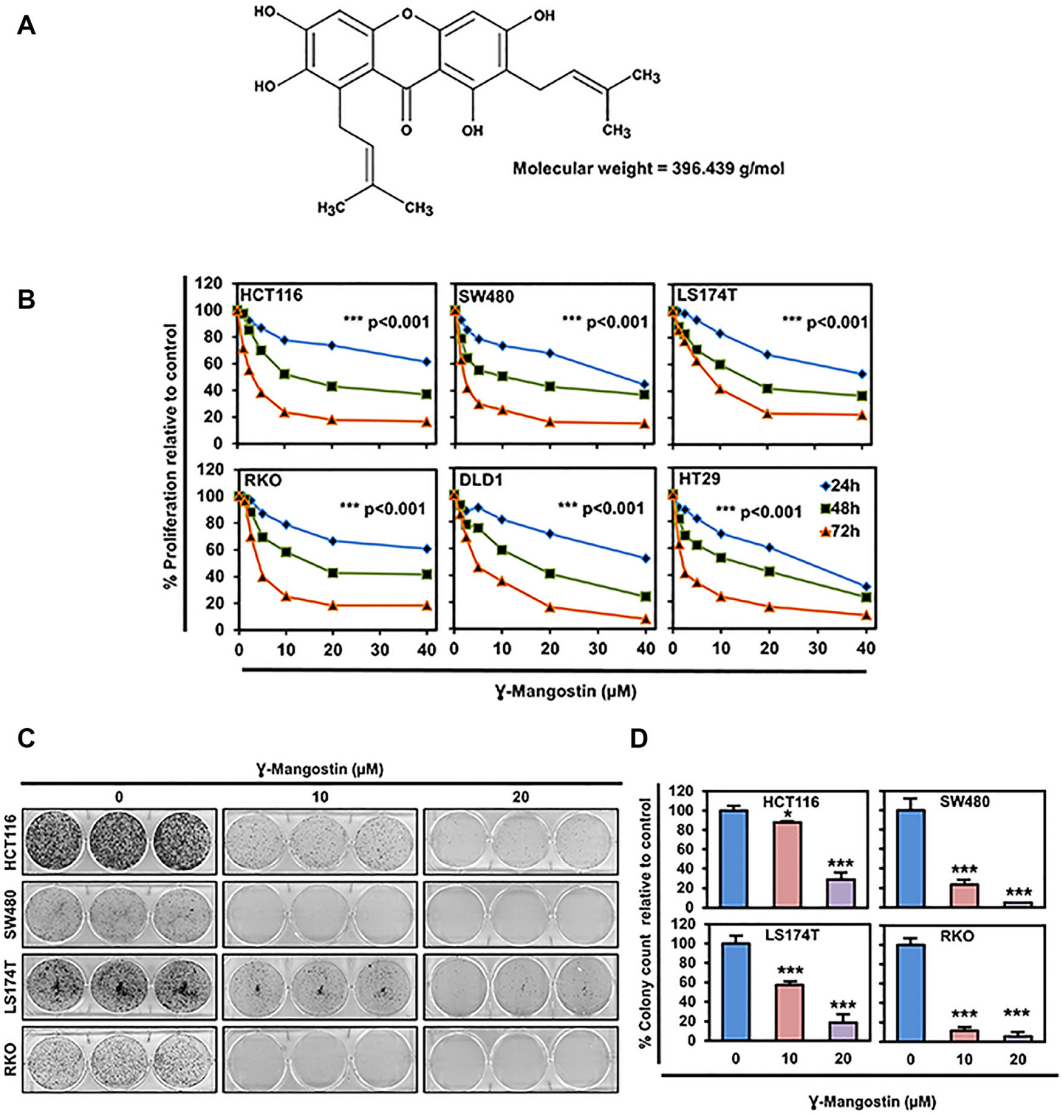
γ-Mangostin inhibits colon cancer cell proliferation. **(A)** Chemical structure of γ-Mangostin. **(B)** γ-Mangostin inhibits proliferation of colon cancer cells. Cells were incubated with increasing dose of γ-Mangostin (0-40 µM) for upto 72 h and assessed for cell proliferation. γ-Mangostin treatment resulted in a significant reduction in cell proliferation with increase in concentration and time when compared to the untreated controls (***p<0.001, 0 vs 5- 40 µM for up to 72 h). **(C)** γ-Mangostin inhibits colony formation. γ-Mangostin (0-20µM) was incubated with colon cancer cells for 48 h and allowed to grow as colonies for 10 days. Incubation with γ-Mangostin inhibits colony formation. **(D)** γ-Mangostin inhibits colon cancer cell colony number. Colony formation of Colon cancer cell lines HCT116, SW480, LS174T and RKO treated with γ-Mangostin were counted using Image J (Fiji) Analysis software. γ-Mangostin treatment significantly inhibits colony number respective to controls (*p<0.05 and ***p<0.001).

Here, we focus on the natural compound γ-Mangostin (Figure 1A), which is a major bioactive compound present in the fruit of Mangosteen (*Garcinia mangostana*), and has been shown to possess anti-cancer activity [10]. Xanthones are the major secondary metabolites present in the mangosteen fruit. All the three derivatives α, β and γ-Mangostin have xanthone as the backbone in their structures. Mangosteen, ‘the queen of fruits’ has been already reported for its anti-bacterial [11–13], antimalarial [14, 15], anticancer [16–21], antioxidant [22, 23] and anti-fungal properties [24, 25]. Here, we aimed to determine the effects of γ-Mangostin on colon cancer growth and elucidated the mechanism, which we have identified to be inhibiting the β-catenin-TCF4 interaction by inhibiting the transcriptional activity of TCF4.

## RESULTS

### γ-Mangostin inhibits cell proliferation and colony formation

We determined the effects of γ-Mangostin (Figure 1A) on six different colon cancer cell lines, HCT116, SW480, LS174T, RKO, HT29 and DLD1. The rationale for choosing these cell lines are due to harboring different types of mutations [26]. γ-Mangostin treatment significantly inhibited the cell proliferation in concentration and time-dependent manner (p<0.001, Figure 1B). The 50% inhibitory concentration values at 48 hours were ranging from 10-15 µM for colon cancer cell lines. The IC_50_ values are listed in Supplementary Table 1. The long-term effect of γ-Mangostin was assessed by colony formation assay (Figure 1C). The cells were treated with different concentrations of γ-Mangostin for 48 hours and then grown in normal medium. γ-Mangostin treatment also inhibited the colony number in the colon cancer cell lines (p<0.05, Figure 1D). Colony formation assay confirmed that the inhibition induced by γ-Mangostin was irreversible.

### γ-Mangostin induces apoptosis

**Figure 2:**
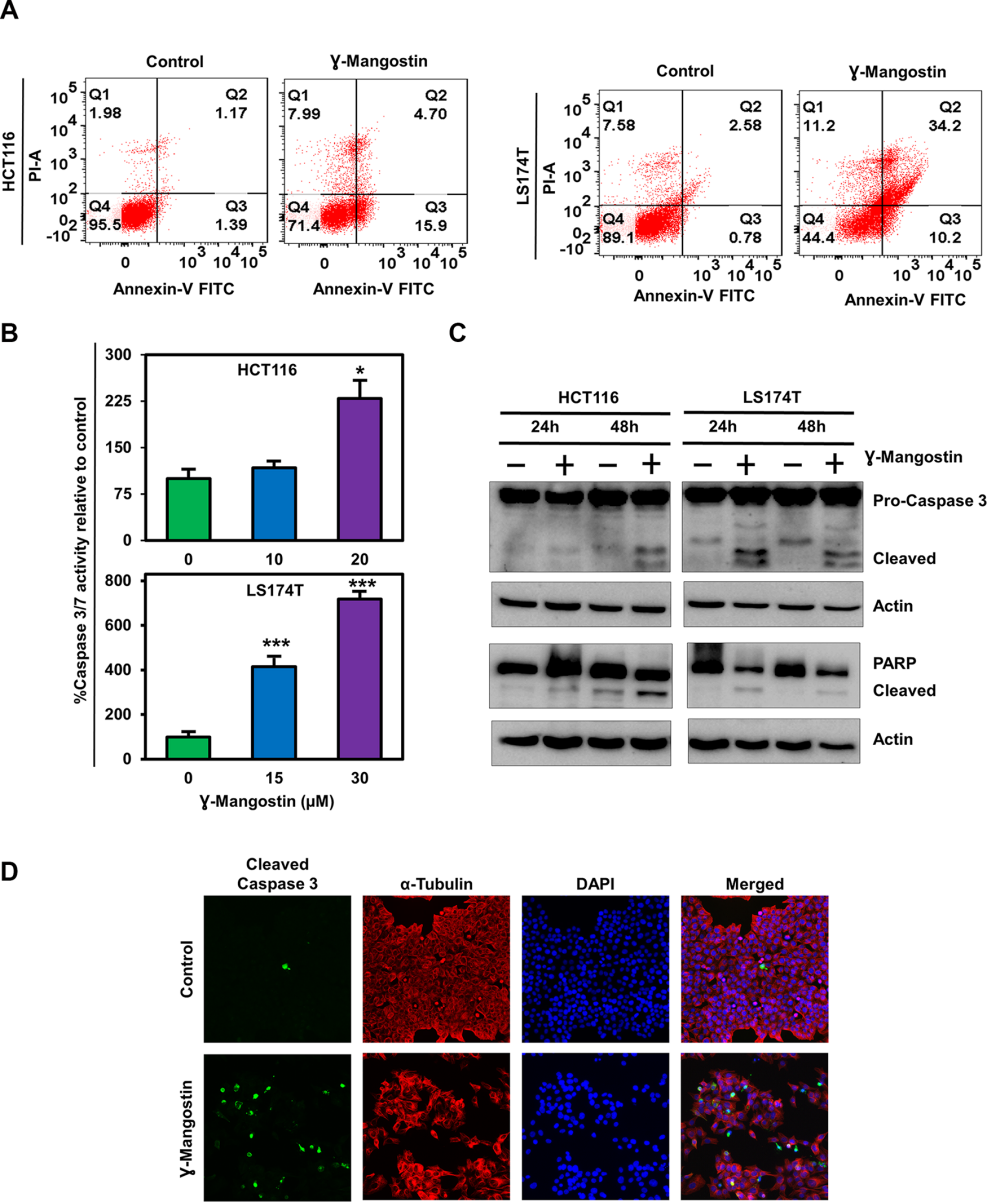
γ-Mangostin induces apoptosis. **(A)** Cells were incubated with γ-Mangostin for 48h and assessed for apoptosis by Annexin V/PI staining. The following are the representations, Q1: Necrosis, Q2: Late apoptosis, Q3: Early apoptosis, Q4: Live cells. γ-Mangostin treatment induces apoptosis in HCT116 and LS174T cells. **(B)** Cells were incubated with γ-Mangostin for 24 h and assessed for apoptosis by Caspase3/7 assay. γ-Mangostin treatment results in significant increase in Caspase3/7 activity in HCT116 and LS174T cells (*p<0.05 and ***p<0.001). **(C)** γ-Mangostin treated HCT116 (10 µM) and LS174T (15 µM) cell lysates were analyzed for Caspase 3 and PARP expression. Both the cell lines showed cleaved Caspase 3 and cleaved PARP expression after γ-Mangostin treatment. **(D)** Immunofluorescence of HCT116 cells, untreated (top) and treated with 10 µM γ-Mangostin (bottom) were evaluated for Cleaved Caspase 3 (green), α-Tubulin (Red) staining with DAPI mounting. Treatment induces increased cleaved caspase 3 expression in γ-Mangostin treatment.

Next, we determined if γ-Mangostin induces apoptosis in HCT116 and LS174T cell lines by the Annexin V/propidium iodide (PI) staining (Figure 2A). Of interest, γ-Mangostin treatment induced 15.9% of early apoptosis and 4.7% of late apoptosis in HCT116 cells, whereas in LS174T cells the values were 10.2% for early apoptosis and 34.2% for late apoptosis. These results were further confirmed by caspase 3/7 assay. γ-Mangostin treatment significantly induced the caspase 3/7 activity in both HCT116 (p<0.05) and LS174T (p<0.001) cell lines at 24 h (Figure 2B). Western blot analysis also revealed an increase in cleaved caspase 3 and cleaved PARP levels after γ-Mangostin treatment at 24 and 48 hours (Figure 2C). Increased cleaved caspase 3 in γ-Mangostin treatment was also visualized by immunofluorescence (Figure 2D). These data collectively suggest that γ-Mangostin treatment induces apoptosis-mediated cell death.

### γ-Mangostin induces cell cycle arrest

**Figure 3:**
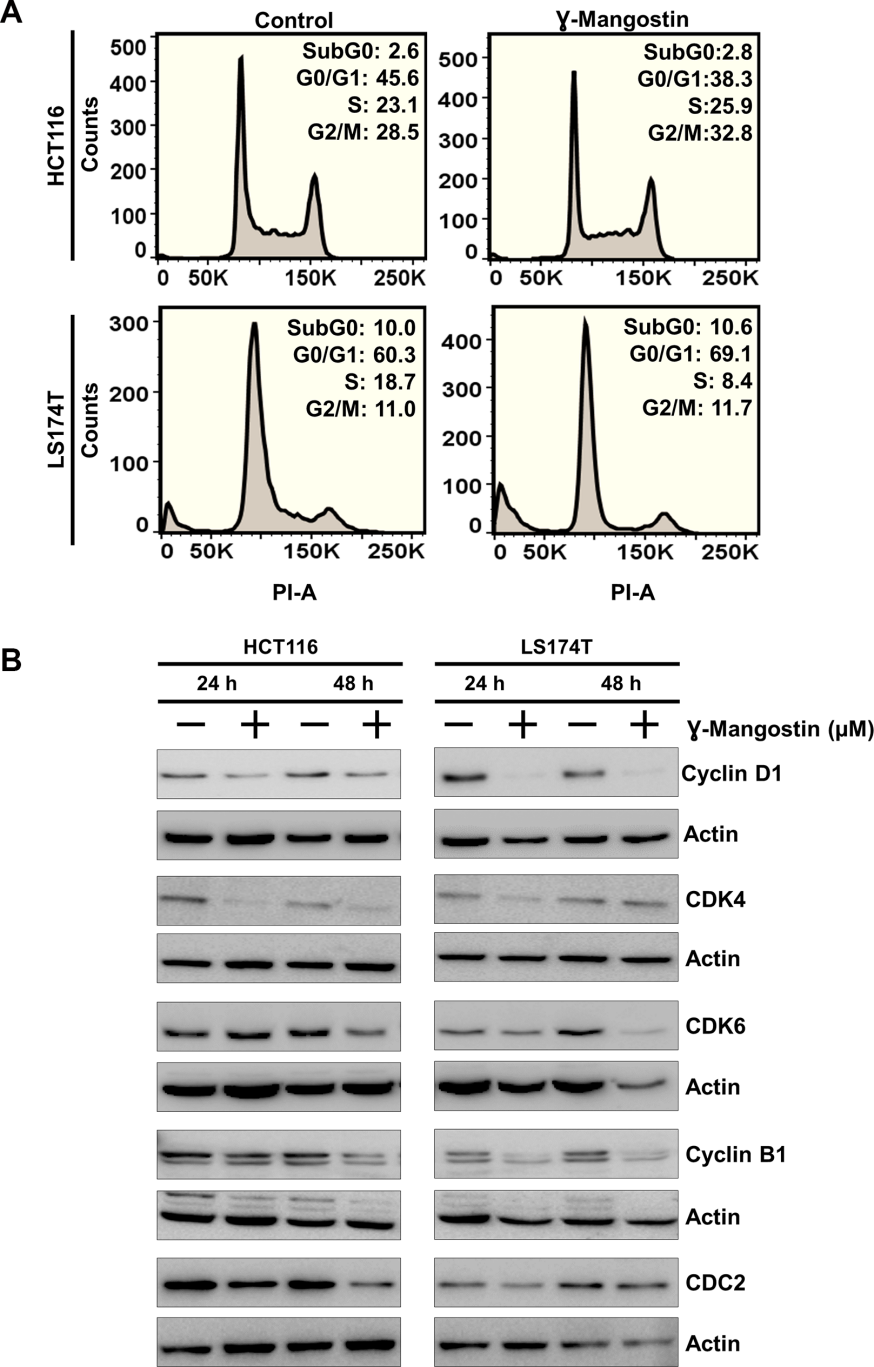
γ-Mangostin inhibits cell cycle progression. **(A)** γ-Mangostin treated HCT116 (10 µM) and LS174T (15 µM) cells were analyzed by flow cytometry. γ-Mangostin treatment induces G2/M and G0/G1 arrest in HCT116 and LS174T cells respectively. **(B)** γ-Mangostin treated HCT116 (10 µM) and LS174T (15 µM) cell lysates were analyzed for Cyclin D1, CDK4, CDK6, Cyclin B1 and CDC2 expression. Treatment shows reduction in the expression levels of Cyclin D1, CDK4, CDK6, Cyclin B1 and CDC2.

Considering that γ-Mangostin inhibits cell proliferation and induces apoptosis, we further evaluated its effect on cell cycle progression. γ-Mangostin treatment induced HCT116 cells to undergo G2/M cell cycle arrest at 48 hours following treatment. However, in LS174T cells, γ-Mangostin treatment induced G0/G1 phase cell cycle arrest (Figure 3A). This was further confirmed by expression of specific cell cycle marker proteins. Western blot analysis revealed a reduction in in cyclin D1 was observed along with CDK4 and CDK6 expression which are markers for the G0/G1 phase. Further, a reduction cyclin B1 and cdc2 expression in both the cells lines which is a marker for G2/M phase (Figure 3B). Cyclin D1 downregulation was more pronounced in LS174T cells. These results confirmed that γ-Mangostin treatment induces cell cycle arrest in colon cancer cell lines thereby inducing cell death.

### γ-Mangostin suppresses Wnt/β-catenin pathway

**Figure 4:**
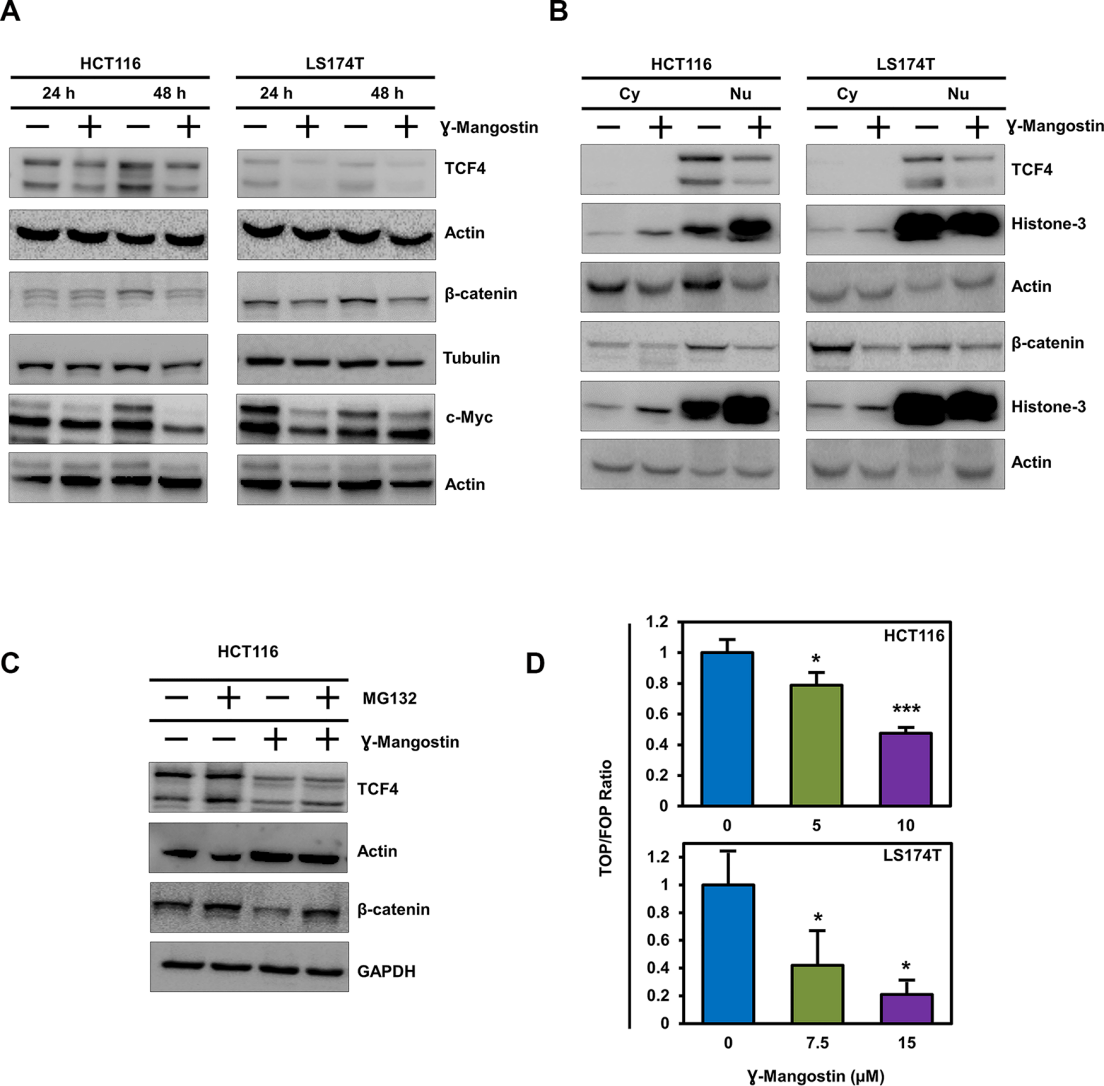
γ-Mangostin inhibits TCF4 expression. **(A)** γ-Mangostin treated HCT116 (10 µM) and LS174T (15 µM) cell lysates were analyzed for TCF4, total β-catenin and c-Myc expression. Treatment shows reduction in the expression levels of TCF4, total β-catenin and c-Myc. **(B)** Nuclear/ Cytoplasmic extracts of HCT116 and LS174T cells treated with γ-Mangostin showed decrease TCF4 expression (nuclear extract) and total β-catenin (both nuclear and cytoplasmic extracts). **(C)** For the proteasomal degradation experiment, HCT116 cells was treated with γ-Mangostin for 48 hours. MG132 was added six hours before collecting the lysate. Western blot analysis showed decrease in the TCF4 and total β-catenin expression after treatment. MG132 added γ-Mangostin treated cells showed increased expression when compared to the treated cells alone. **(D)** HCT116 and LS174T cells were transfected with 450 ng of Super 8x TOPFlash or FOPFlash luciferase plasmid and 50 ng of *pRL-null* vector for 4 hours. Cells were then treated with γ-Mangostin for 24 hours and analyzed by Dual-luciferase assay kit. Treatment inhibits TCF reporter activity in both HCT116 and LS174T cells (*p<0.05 and ***p<0.001).

The Wnt/β-catenin signaling pathway activates transcription of many genes involved in cancer cell proliferation, including cell cycle regulated gene cyclin D1 [27]. Moreover, β-catenin has been shown to increase during S-phase with maximum levels during G2/M [28]. Wnt signaling is active when β-catenin binds to TCF4 and together induce transcription of target genes such as cyclin D1 an c-myc [27, 29]. We first determined the expression of β-catenin and TCF4 following γ-Mangostin treatment. Treatment with the compound resulted in significant reduction in the expression of the two proteins and its downstream targets c-myc (Figure 4A). We also performed western blot analyses to access the levels of β-catenin and TCF4 in the cytoplasmic and nuclear fractions. γ-Mangostin treatment also significantly reduced nuclear β-catenin and TCF4 levels in HCT116 and LS174T cell lines. Moreover, the treatment also reduced cytoplasmic β-catenin expression (Figure 4B). β-catenin and TCF4 levels have been shown to be affected by proteosomal degradation [30, 31]. Accordingly, to determine whether β-catenin and TCF4 is affected by degradation, we used the proteosomal inhibitor MG132 (10 µM for 6 h). Western blot analysis showed that addition of MG132 suppressed γ-Mangostin reduction in the levels of two proteins (Figure 4C). Next, we evaluated the ability of β-catenin-TCF4 interaction to activate transcription following binding to its cognate DNA binding site. For this, we performed the TOP/FOP assay, where TOP Flash encodes the firefly luciferase reporter under the control of a minimal promoter and TCF4 binding sites. Other the other hand, FOP Flash is the control which encodes everything that TOP Flash has except the TCF4 binding site, and therefore is not responsive to β-catenin-TCF4 activity. γ-Mangostin significantly inhibited the TOP Flash luciferase reporter activity in both HCT116 (p<0.001) and LS174T (p<0.05) cell lines (Figure 4D). These data suggest that γ-Mangostin inhibits β-catenin-TCF4 activity, thereby reducing cell growth.

### γ-Mangostin binds TCF4, and not β-catenin protein

**Figure 5:**
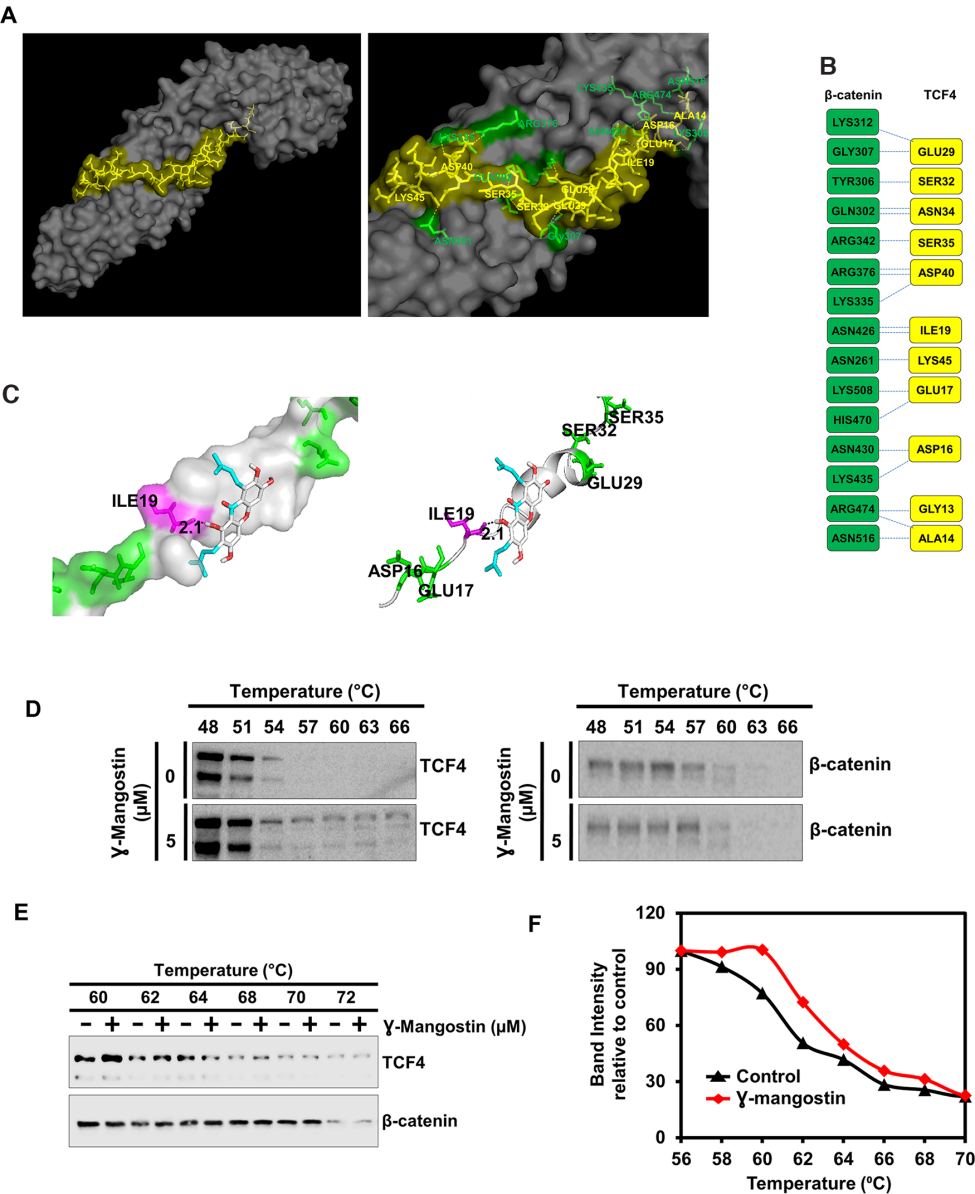
γ-Mangostin interacts with the β-catenin/TCF4 complex. **(A)** Left panel shows structure of β-catenin/TCF4 complex (PDB code: 1JDH) and right panel shows the amino acid interactions of β-catenin (Green) and TCF4 (Yellow) structure. **(B)** Protein-protein interface of β-catenin (Green) and TCF4 (Yellow) structure. Residue interactions by hydrogen bonds (blue dotted line) across interface were shown. **(C)** Molecular docking study shows γ-Mangostin binds to TCF4 protein in beta-catenin binding site (Left panel: Cartoon view, right panel: surface view). γ-Mangostin interacts with Ile-19 (2.1 Å) of TCF4 with the binding energy of -5.5 Kcal/mol. **(D)** Cellular thermal shift assay (CETSA)-Method I. HCT116 cells were treated with γ-Mangostin (5 µM) and subjected to differential temperature treatment for 3 mins. Resulting lysates were subjected to western blot analysis. γ-Mangostin protected TCF4 against thermal degradation, suggesting that the compound interacts with the receptor. **(E-F)** Cellular thermal shift assay (CETSA)-Method II. HCT116 cell lysates were collected and treated with γ-Mangostin (20 µM) and subjected to differential temperature treatment for 3 mins. Resulting lysates were subjected to western blot analysis. γ-Mangostin protected TCF4 at 60 ºC and 62ºC against thermal degradation, again suggesting that the compound interacts with the receptor.

To determine the mechanism by which γ-Mangostin affects β-catenin-TCF4 activity, we performed molecular docking studies. We first determined the protein structure of β-catenin binding to TCF4 along with the interacting amino acids by molecular modeling (Figure 5A). This data looks similar to the published X-ray crystallographic structure [32]. Protein-protein interface of β-catenin and TCF4 are shown in Figure 5B. We also screened for target engagement of γ-Mangostin and determined that the compound interacts with TCF4 (Figure 5C). Our data suggested that γ-Mangostin binds to TCF4 with the binding energy of -5.5 Kcal/mol, the interaction site being isoleucine-19 (ILE-19) (Figure 5C). To experimentally confirm that γ-Mangostin interacts with TCF4 and not β-catenin, we performed the cellular thermal shift assay (CETSA). For this, we treated HCT116 cells with γ-Mangostin, which resulted in a shift in the denaturation temperature of TCF4 compared to the control cells (Figure 5D). However, there was no stabilization observed for β-catenin between the control and γ-Mangostin treatment (Figure 5D). Further, we confirmed these results by performing CETSA by using HCT116 cell lysates (Figure 5E–5F). These data indicate that γ-Mangostin interacts with the TCF4, thereby inhibiting the β-catenin/TCF4 interaction.

### γ-Mangostin inhibits colonosphere formation and expression of stem cell markers

**Figure 6:**
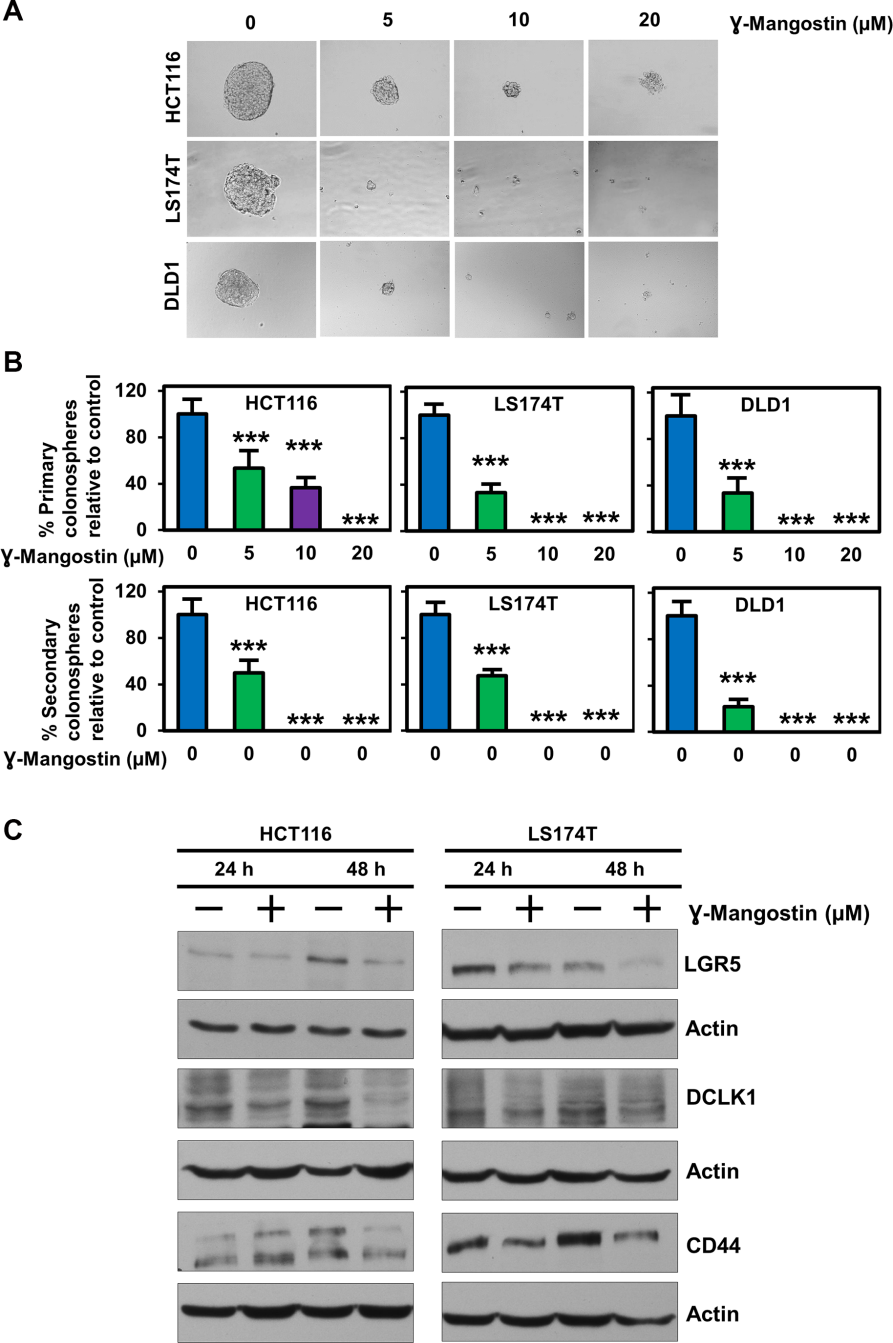
γ-Mangostin inhibits colonosphere formation and stem cell marker expression. **(A)** HCT116, LS174T and DLD1 cells were grown in ultra-low attachment plates and treated with γ-Mangostin of increasing concentration. After 5 days the spheroids were imaged and the numbers were counted. γ-Mangostin significantly inhibits spheroid growth in all the three cell lines with increasing concentration. **(B)** Primary spheroids number were calculated with relative to control. γ-Mangostin significantly inhibits the primary spheroid number. The primary spheroids were dissociated into single cells and re-plated for secondary spheroids. The secondary spheroids were allowed to grow for 5 days and counted. γ-Mangostin significantly inhibits the secondary spheroid number (*p<0.05 and ***p<0.001). **(C)** γ-Mangostin treated HCT116 (10 µM) and LS174T (15 µM) cell lysates were analyzed for LGR5, DCLK1 and CD44 expression. Treatment shows reduction in the expression of LGR5, DCLK1 and CD44.

Since stem cells confer on tumor cells to grow in suspension, we assessed the effect of γ-Mangostin in HCT116, LS174T and DLD1 cell lines. Colonospheres were grown in a serum free media containing stem cell growth factors required to form an intact spheroid. γ-Mangostin treatment inhibited colonosphere formation (p<0.001, Figure 6A), with significant reduction in the number of both first generation (primary) and second generation (secondary) spheroids (p<0.001, Figure 6B). We also evauated the expression of specific stem cell markers LGR5, DCLK1 and CD44. γ-Mangostin treatment significantly inhibited the expression of these stem cell markers (Figure 6C). These data suggest that γ-Mangostin inhibits cancer stem cell marker protein affecting the colonosphere formation.

### γ-Mangostin inhibits tumor xenograft growth

**Figure 7:**
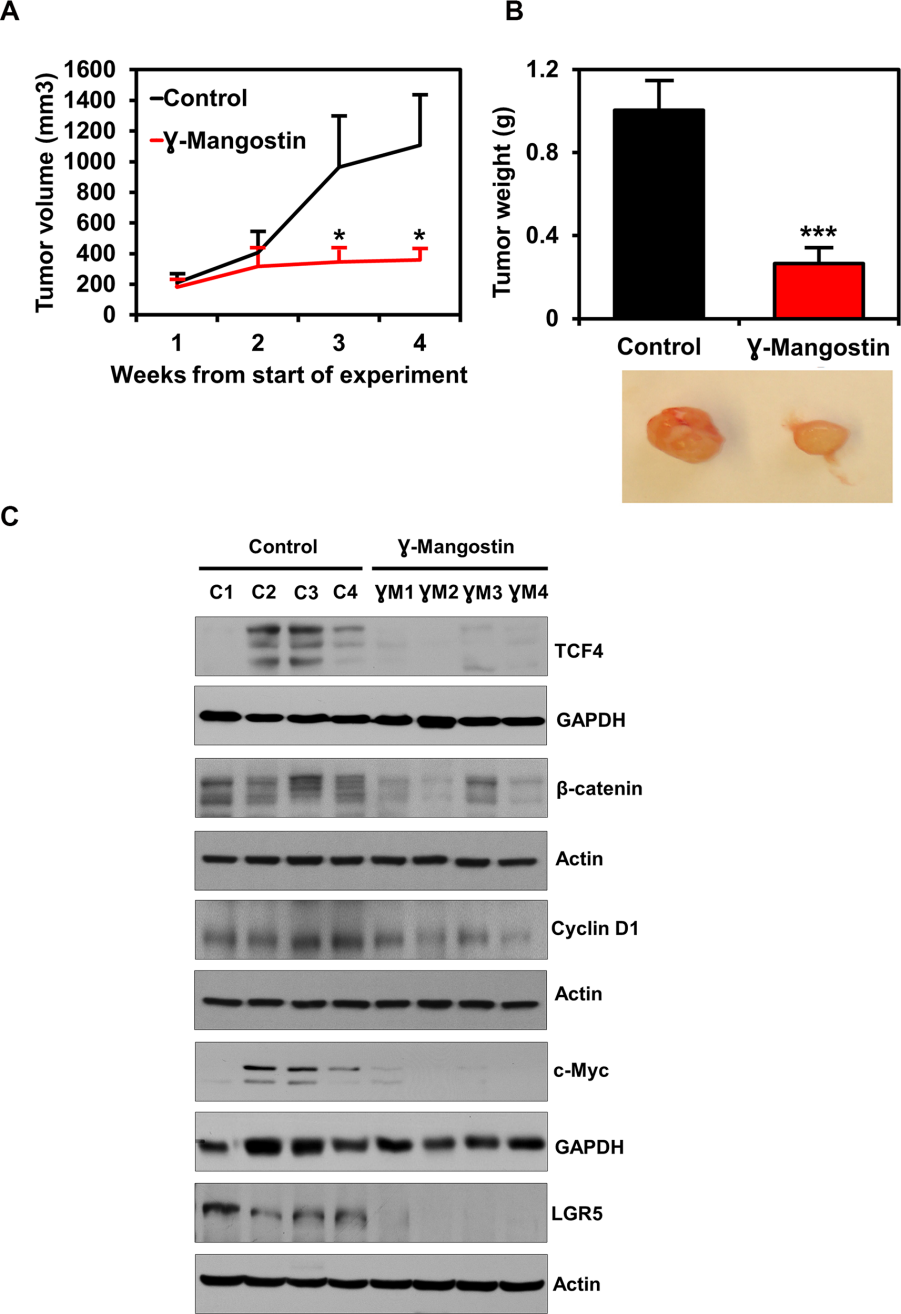
γ-Mangostin inhibits tumor xenograft growth. **(A)** Athymic nude mice were injected with 1×10^6^ HCT116 cells into the flank and allowed to grow into tumors. γ-Mangostin (5 mg/kg body weight) was administered intraperitoneally daily for 21 days. γ-Mangostin treatment induces significant reduction in tumor weight compared to the control (*P<0.05). **(B)** Tumor volume was measured every week. γ-Mangostin treatment results in significant reduction in tumor volume compared to the control (***p<0.001). **(C)** Western blot analysis in γ-Mangostin treated tumor tissue samples showed decrease TCF4, total β-catenin, Cyclin D1, c-Myc and LGR5 expression compared to the control group tumor tissues.

To determine the effect of γ-Mangostin on tumor growth *in vivo*, nude mice harboring HCT116 tumor xenografts in their flanks were administered 5 mg/Kg of γ-Mangostin intraperitoneally for 21 days. γ-Mangostin treatment significantly reduced the tumor growth (p<0.05, Figure 7A), with notably lower tumor volume and weight (p<0.001, Figure 7B). Further, western blot analysis revealed a significant decrease in the expression of TCF4, β-catenin and its downstream targets Cyclin D1, c-myc (Figure 7C). The treatment also decreased expression of LGR5, a stem cell marker that is a target of the Wnt signaling pathway. These data suggest that γ-Mangostin has the ability to inhibit tumor growth.

## DISCUSSION

Colon cancer exerts a staggering effect on healthcare, ranking third in the incidence and mortality in both men and women in the United States [33]. 5-fluorouracil (5-FU) and capecitabine, are the current standard chemotherapy for colon cancer treatment [34] with low response rates of 10–15% [35]. Sporadic colon cancers are mostly caused by genetic mutations of APC and β-catenin which results in β-catenin stabilization and formation of the β-catenin/TCF4 complex. There are numerous Wnt signaling pathway inhibitors developed including targeting β-catenin:TCF4 interactions, however, while some of them may show promise, none of them have been approved by the FDA [8, 9]. There is also a need to show binding to TCF4 to inhibit β-catenin:TCF4 interactions, hence the need to develop an inhibitor that binds TCF4 and inhibits β-catenin:TCF4 interaction remains a challenge. We have previously reported the role of different natural compounds in inhibiting the colon cancer growth [36–38]. Previous work on γ-Mangostin demonstrated the inhibition of β-catenin was through the increased expression of PKG, cGMP dependent kinase and cGMP, a PKG activator which involves the non-classical Wnt/β-catenin signaling [10]. In the present study, we have deciphered the role of γ-Mangostin in inhibiting the colon cancer growth by targeting TCF4, thereby hindering β-catenin:TCF4 interaction. We have employed *in silico* approach along with thermal denaturation analysis to show the interaction of γ-Mangostin with TCF4. Additionally, we have also carried out tumor xenograft experiments to study the efficacy of γ-Mangostin, *in vivo*.

In our study we have shown that γ-Mangostin inhibits cell proliferation and colony formation in the colon cancer cells. The inhibition of cell proliferation by α and γ-Mangostin have been reported in various cancers including melanoma, brain, breast, prostate, colon and pancreas [39]. Our study also showed that γ-Mangostin induces apoptosis and cell cycle arrest in colon cancer cells. Similarly, α and γ-Mangostin was shown to induce apoptosis and G1 phase cell cycle arrest in melanoma cells [39]. Further, the induction of apoptosis and cell cycle arrest has been reported in breast cancer cells [39]. α-mangostin treatment also induced apoptosis and cell cycle arrest in pancreatic cancer cells [39]. One interesting point is that we observed HCT116 cells undergoing G2/M phase cell cycle arrest, whereas LS174T showed G0/G1 cell cycle arrest. It has been reported that β-catenin/TCF4 complex formation is high in S and G2 phase of the cell cycle and this plays a significant role in transcribing the Wnt target genes [40]. However, inhibiting this interaction decrease the cell proliferation and G2/M phase cell cycle progression. It is shown that β-catenin levels are high in S-phase of the cell cycle and reaches the maximum level in the G2/M phase of the cell cycle [28]. Moreover, inhibiting the β-catenin/TCF4 interaction by a diterpenoid derivative, 15-oxospiramilactone (NC043) induces G2M arrest in colon cancer cells [41]. Further studies are required to determine whether unique mutations in the LS174T cell line contributes to G0/G1 arrest, and whether this is also observed in patient samples harboring the same mutation.

In order to induce cytotoxicity in colon cancer cells, targeting the β-catenin:TCF4 interaction is critical. The N-terminal region amino acids of TCF4 that are required for interacting with β-catenin are Ala14, Asp16, Glu17, Ile19, Glu29, Ser32, Asn34, Ser35, Asp40 and Lys45 [42]. This suggests that interaction of β-catenin and TCF4 spread over a large surface area [43]. However, our molecular docking data shows that γ-Mangostin interacts with ILE-19 of TCF4. This suggests that γ-Mangostin possibly binding to a single amino action in TCF4 is sufficient enough to inhibit TCF4:β-catenin. This was confirmed by CETSA assay, where γ-Mangostin stabilized TCF4 but not β-catenin. Further, a reporter assay confirmed a reduction in the transcriptional activity of β-catenin:TCF4 following γ-Mangostin treatment. Other studies also reported that targeting the β-catenin/TCF4 interaction suppresses the β-catenin/TCF4 driver reporter activity [44, 45], followed by a decrease in cell viability and clonogenicity. Moreover, it has been shown in colon cancer that the knockdown of TCF4 rather than knockdown of β-catenin enhances the chemosensitivity of the drugs in inhibiting the cell proliferation and inducing apoptosis [46].

Dynamic transcriptional activation of the β-catenin promoter results in increased nuclear β-catenin levels where it interacts with the TCF/LEF transcription factors and activates the β-catenin:TCF target genes [47]. We have now shown that γ-Mangostin not only inhibits the nuclear fraction of β-catenin and TCF4, but it also inhibits the β-catenin expression in the cytoplasm. Our data also shows the proteasomal degradation of TCF4 in HCT116 cells after γ-Mangostin treatment. Proteasomal degradation of TCF4 has been reported earlier in colon cancer cells after resveratrol treatment, followed by decreased TCF4 protein expression [31].

β-catenin and TCF4 interaction can also influence stemness signature genes in colon cancer cells [48]. One such protein is LGR5. Numerous reports have demonstrated LGR5 overexpression in colon cancer, and this correlates with increased proliferation and chemoresistance [49–52]. LGR5 positive cells in the tumor has been reported to have increased TCF/LEF activity along with increased clonogenic potential both *in vitro* and *in vivo* [53]. Further in our previous studies, we have shown that DCLK1 acts as stem cell marker in cancer cells as well as a quiescent marker in normal cells of the intestine [54, 55]. Here we have shown that γ-Mangostin decreases expression of both LGR5 and DCLK1 in colon cancer cells along with inhibition of colonosphere formation. This clearly shows that γ-Mangostin also affect the stemness property in colon cancer cells by inhibiting the β-catenin/TCF4 interaction. Furthermore, downstream targets of the β-catenin/TCF4 complex such as cyclin D1 and c-myc were also reduced following γ-Mangostin treatment. This was also observed *in vivo* in HCT116 tumor xenograft model which showed a reduction in TCF4, β-catenin and LGR5 expression after γ-Mangostin treatment along with the reduced tumor volume and tumor weight.

In conclusion, our study has deciphered a target of γ-Mangostin through inhibition of the β-catenin/TCF4 complex formation. γ-Mangostin may be a potential therapeutic target for colon cancer. Future studies will focus on further developing preclinical data that will enable moving the compound to the clinic either in the neo adjuvant or adjuvant setting. In addition, our studies will utilize a medicinal chemistry approach to develop novel analogs γ-Mangostin that has better efficacy at lower concentrations in inhibiting TCF4:β-catenin interactions to suppress tumorigenesis.

## MATERIALS AND METHODS

### Cells and reagents

HCT116, SW480, RKO, DLD1, HT29 and LS174T (American Type Culture Collection, Manassas, VA) cell lines were grown in DMEM medium (Corning, Tewksbury, MA) containing 10% fetal bovine serum (VWR, Radnor, PA) and 1% antibiotic-antimycotic solution (Corning, Tewksbury, MA) at 37°C in a humidified atmosphere containing 5% CO2. The cells used were within 20 passages after reviving. STR allele profiling was performed to authenticate the cell lines by an independent source (NIH-funded University of Arizona Genetics Core, Tucson, AZ-Cell Line Authentication Core). The validity of the cell lines was confirmed by 80% homology to published STR profiles. γ-Mangostin was purchased from (Sigma-Aldrich, St. Louis, MO).

### Proliferation and clonogenicity assay

Cells were grown in 96 well plates and treated with different concentration of γ-Mangostin. Proliferation was analyzed by a hexoseaminidase assay. For clonogenicity assay, six-well plates were seeded with 500 viable cells per well and allowed to grow for 24 h. The cells were then incubated in the presence of varying concentrations of γ-Mangostin for 48 h. γ-Mangostin containing medium was then removed, and the cells were rinsed in phosphate buffered saline (PBS) and incubated for an additional 10 days in complete medium. The colonies obtained were washed with PBS and fixed in 10% formalin for 10 minutes at room temperature, followed by washing with PBS and staining with Crystal violet. The colonies were counted using Image J (Fiji) software. The colonies were then compared with untreated cells [56].

### Caspase 3/7 assay

HCT116 and LS174T cells grown in 96 well plates were treated with γ-Mangostin for 24. Caspase 3/7 activity was examined using the Caspase-Glo 3/7 Assay systems according to the manufacturer’s instructions (Promega, Madison, WI).

### Cell cycle analysis and Annexin V/PI staining

HCT116 and LS174T cells were treated with γ-Mangostin for 24 h, then cells were trypsinized and resuspended in PBS. Single-cell suspensions were fixed using 70% ethanol for 2 h, and subsequently permeabilized and stained with FxCycle PI/RNase staining solution (Thermo Fisher Scientific, Waltham, MA) at room temperature. Flow cytometry was done with a BD LSR II (BD Biosciences) capturing 10,000 events for each sample. For Annexin V/PI staining, flow cytometry was performed using dead cell apoptosis kit with Annexin V FITC and PI solution (Thermo Fisher Scientific, Waltham, MA) after treatment with γ-Mangostin. Results were analyzed with FlowJo software.

### Western blot analysis

Whole cell lysates were prepared with RIPA buffer and then subjected to polyacrylamide gel electrophoresis and blotting onto Immobilon polyvinylidene difluoride membranes. Specific proteins were detected by incubating the membranes with respective primary antibody and suitable HRP-conjugated secondary antibody which then detected by the enhanced chemiluminescence system. For the nuclear-cytoplasmic extracts, the cells were extracted using 0.1% NP40 (Thermo Fisher Scientific Waltham, MA) and the cytoplasmic and nuclear fractions were collected separately. For the proteasomal degradation experiment, HCT116 cells were treated with γ-Mangostin for 48 hours. MG132 (10 µM) (Sigma-Aldrich, St. Louis, MO) was added six hours before collecting the lysate.

### Cellular thermal shift assay (CETSA)

CETSA analysis was performed by two methods. In Method I HCT116 cells were treated with γ-Mangostin containing media for 4 hours and subjected to differential temperature treatment (42-72ºC) for 3 mins. In Method II, protein lysates were prepared from HCT116 cells and the lysates were incubated with γ-Mangostin for 2 hours. The resulting lysates were subjected differential temperature treatment (42–72ºC) on thermal cycler, centrifuged and subjected to western blot analysis [57, 58].

### TOP/FOP flash assay

M50 Super 8x TOPFlash (Addgene plasmid # 12456; https://www.addgene.org/12456/:12456; RRID: Addgene_12456) and M51 Super 8x FOPFlash (TOPFlash mutant) was a gift from Randall Moon (Addgene plasmid # 12457; https://www.addgene.org/12457/:12457; RRID:Addgene_2457) [59]. HCT116 and LS174T cells were plated in 24-well plates and transfected with plasmid mixtures containing 450 ng of Super 8x TOPFlash or FOPFlash plasmid and 50 ng of a *pRL-null* vector (Promega, Madison, WI) for 4 h at 37 °C under a humidified atmosphere of 5% CO_2_. The transfected cells were treated with γ-Mangostin. Dual-luciferase assay kit (Promega, Madison, WI) was used to measure the luciferase activity.

### Immunofluorescence

Cells were grown on coverslips, treated with γ-Mangostin and then fixed using neutral buffered formalin and permeabilized with 0.3 % Triton X-100. This was then blocked with 1% BSA in PBS and then incubated overnight with primary antibody at 4° C. Suitable fluorophore-conjugated secondary antibody was incubated at room temperature for 1 hour. The coverslips were mounted using VECTASHIELD Antifade Mounting Medium with 4′,6-diamidino-2-phenylindole (DAPI, Vector Laboratories).

### Colonosphere assay

HCT116, LS174T and DLD1 cells were grown in ultra-low attachment plates containing RPMI 1640 with specific growth factors such as EGF, bFGF and B27 supplement. After γ-Mangostin treatment, the spheroids were allowed to grow for 5 days. The spheroids were then imaged and counted. For secondary spheroids, the primary spheroids were dissociated into single cells and re-plated again without γ-Mangostin treatment and counted after 5 days [56].

### HCT116 xenograft tumors in mice

Five-week-old male athymic nude mice (Charles River) were utilized for *in vivo* experiments. They were maintained with water and standard mouse chow ad libidum and used in protocols approved by the Institutional Animal Care and Use. Animals were injected with 1×10^6^ HCT116 cells into the flank and allowed to grow into tumors. Upon the identification of a palpable tumor, γ-Mangostin (5 mg/kg body weight) was administered intraperitoneally once daily for 21 days. At the end of treatment, the animals were euthanized, and the tumors were excised, weighed and subjected to western blot analysis.

### Statistical analysis

The values were expressed as Mean ± SD. One-way ANOVA was used to analyze the data followed by post-Bonferroni test. Student t-test was applied to calculate the weekly tumor volume in xenograft experiment. A *p* value of less than 0.05 was considered as statistically significant.

## SUPPLEMENTARY MATERIALS AND TABLES



## Abbreviations

APC: Adenomatous polyposis coli
β-TrCP: β-Transducin repeats containing protein
CETSA: Cellular thermal shift assay
DAPI: 4′,6-diamidino-2-phenylindole
GSK3β: glycogen synthase kinase 3β
PI: Propidium Iodide
TCF4: T-Cell Factor 4.
