# Supplementary material for: Targeting transcription factor TCF4 by γ-Mangostin, a natural xanthone
Source: Oncotarget. 2019 Sep 24;10(54):5576–91. doi: 10.18632/oncotarget.27159 (PMC6771460; doi:10.18632/oncotarget.27159)
Supplement: Supplementary file 1 [file oncotarget-10-5576-s001.pdf]

Targeting transcription factor TCF4 by γ-Mangostin, a natural xanthone

SUPPLEMENTARY MATERIALS

Supplementary Table 1: IC-50 values of the cell lines treated with γ-Mangostin at 48 h

Cell line	IC_{50} (µM)
HCT116	10
SW480	10
RKO	15
HT29	10
DLD1	15
LS174T	15
